# Ciliary Motility and Ultrastructure in Bronchial Epithelium of Lung Transplant Recipients with Primary Ciliary Dyskinesia

**DOI:** 10.3390/jcm14103439

**Published:** 2025-05-14

**Authors:** Miguel Armengot, Catalina Bancalari, Lidón Carretero-Vilarroig, Rosana Blanco-Máñez, Noelia Muñoz-Fernández, Enrique Cases, José M. Millán, Santiago Almanzo, Teresa Jaijo

**Affiliations:** 1 Department of Otorhinolaryngology, Hospital Universitari i Politècnic La Fe, 46026 Valencia, Spain; miguel.armengot@uv.es (M.A.); noeliamufer@gmail.com (N.M.-F.); santiagoalmanzo@gmail.com (S.A.); 2 Department of Surgery, Faculty of Medicine and Dentistry, University of Valencia, 46010 Valencia, Spain; 3 Center for Biomedical Network Research on Rare Diseases CIBERER), Carlos III Health Institute, 46026 Valencia, Spain; 4 Molecular, Cellular and Genomic Biomedicine Group (BMCG), IIS La Fe, 46026 Valencia, Spain; lidon_carretero@iislafe.es (L.C.-V.); millan_jos@gva.es (J.M.M.); jaijo_ter@gva.es (T.J.); 5 Department of Pathology, University and Polytechnic Hospital La Fe, 46026 Valencia, Spain; roblanma@gmail.com; 6 Department of Pulmonology, University and Polytechnic Hospital La Fe, 46026 Valencia, Spain; cases_enr@gva.es; 7 Genetics Unit, University and Polytechnic Hospital La Fe, 46026 Valencia, Spain

**Keywords:** ciliary motility, ciliary ultrastructure, bronchiectasis, lung transplantation, ciliopathies, Kartagener syndrome

## Abstract

**Background and Objective:** Primary ciliary dyskinesia (PCD) is a rare genetic disorder that affects the mucociliary system, leading to progressive lung damage. This deterioration can result in bronchiectasis, atelectasis, and respiratory failure, necessitating lung transplantation in severe cases. This study aims to assess ciliary motility and ultrastructure in the bronchial epithelium of transplanted lungs in patients with PCD to determine whether mucociliary function is preserved post-transplantation. The findings seek to enhance scientific understanding and provide prognostic insights for these patients. **Materials and Methods:** A prospective observational study was conducted on two patients with PCD and advanced lung disease who underwent bilateral lung transplantation. Nasal and bronchial cilia samples were analyzed using high-speed videomicroscopy and transmission electron microscopy. Follow-up assessments included ciliary function analysis, lung rejection monitoring, and quality-of-life evaluations, with follow-up extending up to 30 months post-transplant. **Results:** Post-transplant evaluations demonstrated normal ciliary motility and ultrastructure in the transplanted lungs throughout the study period (up to 30 months), indicating the long-term preservation of mucociliary function. **Conclusions:** Transplanted lungs in patients with PCD maintain normal bronchial ciliary motility and structure in the long term, suggesting a favorable prognosis for both the graft and the recipient. These findings support the feasibility and long-term effectiveness of lung transplantation in patients with PCD.

## 1. Introduction

Primary ciliary dyskinesia (PCD) is a hereditary disease with an autosomal recessive inheritance pattern, although sporadic cases of dominant and sex-linked inheritance have also been described [1]. Its prevalence is estimated to be approximately 1 in 7500–10,000 live births [2], although it is likely underdiagnosed in the general population. The condition is characterized by cilia immotility or the dyskinesia [3] (abnormal and ineffective ciliary movement) of motile cilia, which, in the case of respiratory cilia, leads to mucus stasis in the airways. This leads to bacterial proliferation and the following associated complications:

(a) Chronic bronchitis with bronchiectasis and progressive deterioration of lung function;
(b) Chronic sinusitis with frequent exacerbations, as well as sinus hypoplasia and persistent pansinusitis;
(c) Secretory otitis media, which can progress to chronic otitis and hearing loss over time, particularly in childhood [4].

The disease presents clinically from birth, with persistent rhinorrhea and a lifelong productive cough with mucopurulent secretions [4]. Neonatal respiratory distress of unknown cause is also common [5]. Approximately 46% of patients exhibit situs inversus, while 12% present with heterotaxy [6], both resulting from the impaired motility of embryonic nodal cilia. Male patients are often infertile due to sperm flagellar immotility, while female patients experience subfertility due to defective ciliary movement in the fallopian tubes, which hinders ovum transport [7]. The diagnosis of PCD is complex and relies on the following [8,9,10]:

(a) Assessment of ciliary motility using high-speed video microscopy DHSV—Digital High-Speed Leica DMI3000B inverted microscope (Leica, Berlin, Germany) in nasal (more accessible) or bronchial epithelial samples. This technique enables a detailed analysis of the ciliary beat pattern. Variations in patterns have been previously associated with specific genetic findings [11]. Also, this study allows for the measurement of ciliary beat frequency (CBF), which typically ranges from 7 to 16 Hz in healthy individuals under physiological conditions [12]. It is important to note that CBF values are influenced by environmental factors such as temperature and humidity;
(b) Evaluation of ciliary ultrastructure through electron microscopy (EM) in the same samples, although no structural abnormalities are detected in 30% of patients;
(c) Genetic analysis, with over 50 genes associated with PCD identified to date, explaining 65–70% of cases [13].

The lack of a functional mucociliary clearance system complicates treatment, as neither medical nor surgical therapies can restore normal nasosinusal or otological physiology, resulting in lifelong disease persistence. At the pulmonary level, progressive lung damage develops, leading to bronchiectasis, atelectasis, and eventually respiratory failure. In some patients, lung transplantation remains the only viable option for survival [14], though its success depends on whether the transplanted lung maintains a permanently functional mucociliary system.

The hypothesis underlying this study posits that the transplanted lung preserves the donor’s normal ciliary function, leading to a favorable prognosis for the recipient by improving lung function and quality of life. Conversely, if mucociliary function is lost over time, the transplanted lung would progressively deteriorate, ultimately leading to respiratory failure.

The primary objective of this research is to determine ciliary motility and ultrastructure in the bronchial epithelium of transplanted lungs in patients with PCD. This will assess whether the mucociliary function of the organ remains preserved despite being implanted in an individual affected by this genetic disorder. The findings aim to contribute to scientific knowledge and provide valuable insights regarding the impact of lung transplantation on mucociliary function and the prognosis of patients with PCD undergoing this procedure.

## 2. Materials and Methods

A descriptive observational study was conducted on two cases of bilateral lung transplantation in patients diagnosed with PCD and severe disease progression. The study population consisted of two female patients (aged 60 and 63 years) with advanced lung deterioration, who met the eligibility criteria for bilateral lung transplantation due to extensive bilateral bronchiectasis and pulmonary function decline confirmed through respiratory tests. Patient selection followed the established lung transplantation protocols [15]. Ethical approval was granted by the local Ethics Committee of Hospital Universitario y Politécnico La Fe (Registry No.: 2022-291-1, approval date: 29 March 2023). Written informed consent was obtained from all participants.

**Figure 1:**
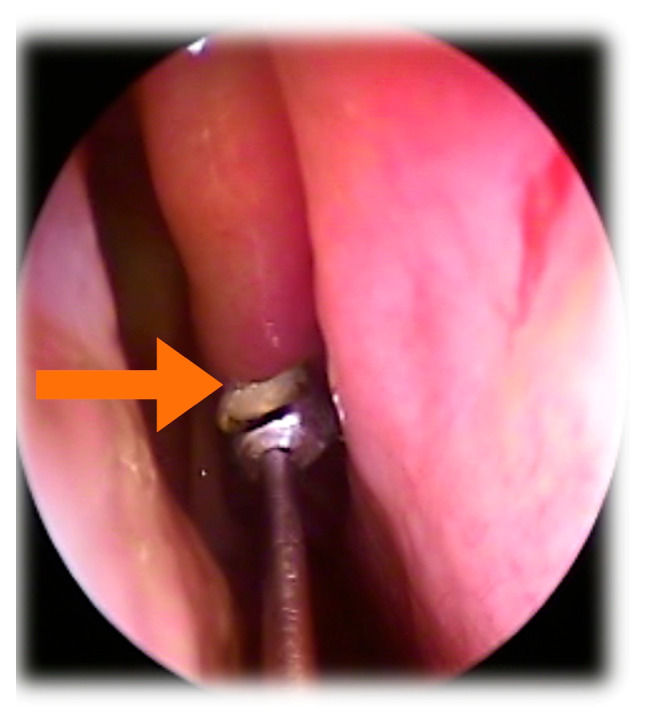
Curettage of middle turbinate mucosa (arrow) under rhinoscopic visualization.

**Figure 2:**
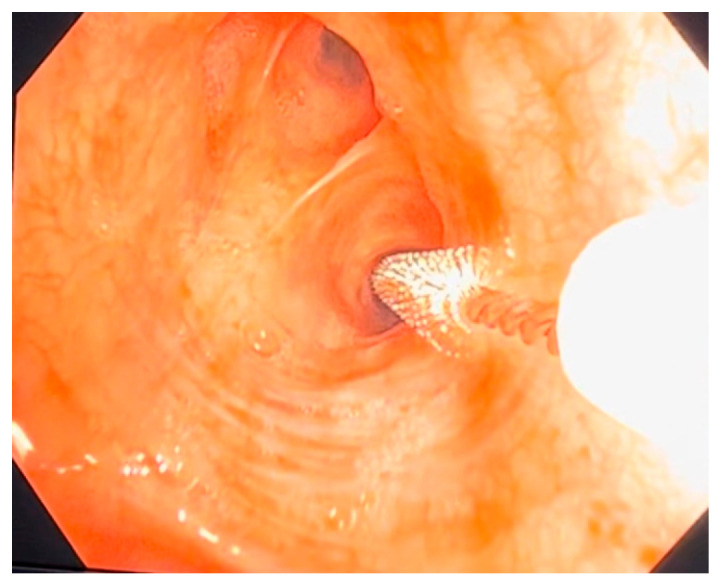
Bronchial brushing performed during bronchoscopy.

**Figure 3:**
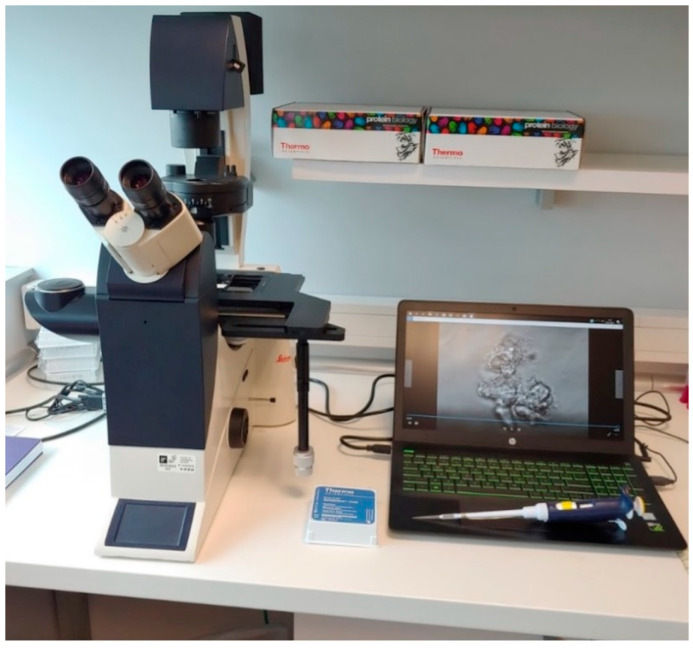
Leica DMI3000B inverted microscope (Berlin Germany) with system for recording and analyzing ciliary motility.

Ciliated cell samples for functional and structural analysis were obtained nasally via curettage of the middle turbinate mucosa under rhinoscopic guidance (Figure 1), while bronchial samples were collected through brushing during bronchoscopy (Figure 2). Cells were collected in DMEM high-glucose medium with streptomycin at 10% to preserve cellular integrity and viability. The ciliary motility of the nasal and bronchial epithelial samples from the transplanted lungs was assessed using high-speed, high-precision videomicroscopy immediately after collection. The temperature was maintained at 24 °C (room temperature). Multiple fields per sample were analyzed to identify regions containing continuous epithelial strips and isolated cells. Images were acquired from both the lateral and top views. The samples were visualized using a Leica DMI3000B inverted microscope (Leica, Berlin, Germany) with a 63× objective lens, providing a total magnification of 630× (Figure 3). Images were recorded using a Basler acA1300-200 um digital video camera with an ON-Semiconductor PYTHON 1300 CMOS sensor (Basler AG, Ahrensburg, Germany) attached to the microscope. All recordings and image processing were performed using the Sisson–Ammons Video Analysis (SAVA) system [16], which allowed for the determination of ciliary motion patterns and ciliary beat frequency (CBF). CBF was measured for each patient throughout the entire follow-up period.

The ciliary ultrastructure of the nasal and bronchial samples from the transplanted lungs was assessed in accordance with the International Consensus Guideline for reporting transmission electron microscopy results in the diagnosis of Primary Ciliary Dyskinesia (BEAT PCD TEM Criteria) (Eur Respir J 2020) [17]. Cells were fixed in 2.5% phosphate-buffered glutaraldehyde (pH 7.3) for 24 h, then gently centrifuged at 1500 rpm for two minutes. The resulting pellet was processed as previously reported [18]. A total of 50 axonemes were analyzed from both the nasal and bronchial ciliated epithelium of the transplanted lungs. Ciliary sections were selected near healthy ciliated cells to minimize the presence of secondary ciliary defects. Transmission electron microscopy (TEM) analysis was performed using a Hitachi HT7700 electron microscope (Hitachi High-Tech Corporation, Tokyo, Japan). Axonemal defects were classified according to the international consensus guidelines.

Patients underwent preoperative evaluations and post-transplant follow-up visits at different time points due to the chronological difference in their transplant years (2021 and 2022, respectively). The first patient was evaluated at 1, 3, 6, 12, 18, 24, and 30 months post-transplant, while the second patient was evaluated at 1, 3, 6, 12, and 18 months. During these visits, variables related to ciliary motility and ultrastructure, as previously described, were recorded and analyzed. Additionally, post-transplant follow-up by the lung transplant unit included pulmonary function tests, imaging studies, blood tests, lung rejection assessments, microbiological cultures, and quality-of-life monitoring.

## 3. Results

The first patient included in this study was a 63-year-old woman who was diagnosed with PCD around the age of 50; however, symptoms associated with the disease had been present since birth. A significant family history finding was that her younger sister had also been diagnosed with PCD. The patient exhibited a compatible clinical phenotype [4], with severe neonatal respiratory distress and chronic symptoms, including productive cough, mucopurulent rhinorrhea, recurrent pneumonias, bronchiectasis, recurrent otitis with tympanic myringosclerosis, and moderate bilateral mixed hearing loss. No laterality defects were observed.

Genetic testing using DNA from peripheral blood identified the following two heterozygous mutations in different genes: *RSPH1* with a nucleotide change c.85G > T and an amino acid change p.Glu29*; and *HYDIN* with a nucleotide change c.6889G > T and an amino acid change p.Glu2297*. These same mutations were present in her sister with PCD.

**Figure 4:**
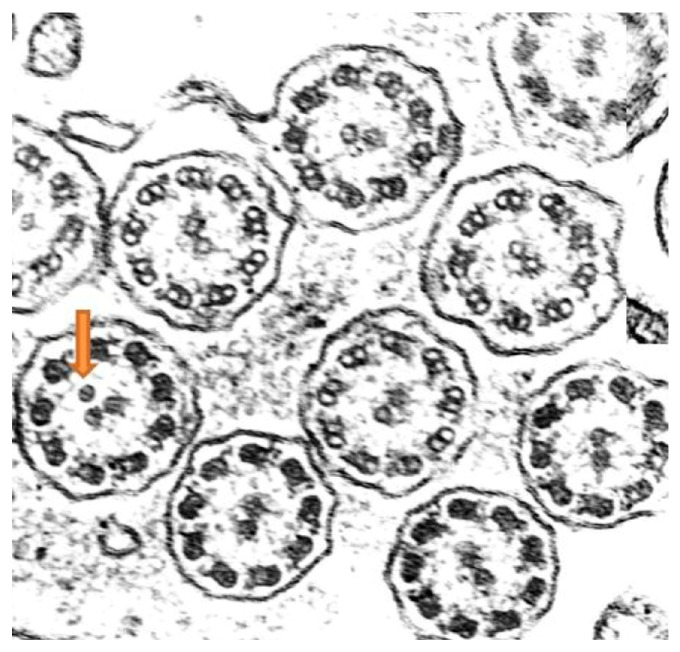
Ciliary sections obtained from first biopsy study (nasal scraping). Predominance of preserved axonemes. Some axonemes present an extra central microtubule (arrow).

Ciliary ultrastructure analysis revealed that both the bronchial and nasal samples preserved normal ultrastructure in 92% of the axonemes, while 8% presented abnormalities, specifically supernumerary microtubules, indicating overall preserved ciliary morphology [19] (Figure 4).

High-speed videomicroscopy analysis of nasal ciliary motility revealed a dyskinetic ciliary movement pattern, primarily characterized by immobile cilia, as well as some residual uncoordinated or vibratory ciliary activity. These findings were consistent with the diagnosis of PCD [3,20].

The second patient was a 60-year-old woman with symptoms present since birth but no known family history of the disease. She exhibited a similar clinical phenotype, with persistent symptoms including productive cough, recurrent otitis with conductive hearing loss, pneumonias, bronchiectasis, atelectasis, and additional findings such as agenesis of the frontal sinus.

Genetic analysis identified the following two heterozygous mutations in the *RSPH1* gene: c.275-2A > C splicing; and c.85G > T with an amino acid change p.Glu29*.

Ciliary ultrastructure analysis showed that 62% of the axonemes maintained normal structure, while 38% presented defects in the central complex, corresponding to a class 2 defect and indicating altered ciliary morphology [19].

Nasal ciliary motility analysis revealed a dyskinetic pattern characterized by limited ciliary movement in a single phase and the presence of rigid cilia (See Supplementary Video S1). Additionally, some cells exhibited completely immobile cilia, consistent with the diagnosis of PCD.

Both patients underwent bilateral lung transplantation due to the unfavorable progression of their condition despite conventional treatments [19], including respiratory physiotherapy, azithromycin three times a week, and inhaled colistin.

Post-transplant ciliary motility and ultrastructure studies followed the same protocol for both patients, although they were conducted in different years according to each transplant date. The sample collection schedule was adapted to each patient’s clinical bronchoscopy requirements. The first patient was assessed at 3, 6, 12, 18, 24, and 30 months post-transplant, showing immotile cilia in the nasal epithelium, whereas the bronchial ciliary function remained normal, displaying the two characteristic phases of coordinated ciliary movement (See Supplementary Video S2). The mean ciliary beat frequency in the bronchial epithelial samples during the follow-up period was 9 Hz, with a range of 8.5–12 Hz.

**Figure 5:**
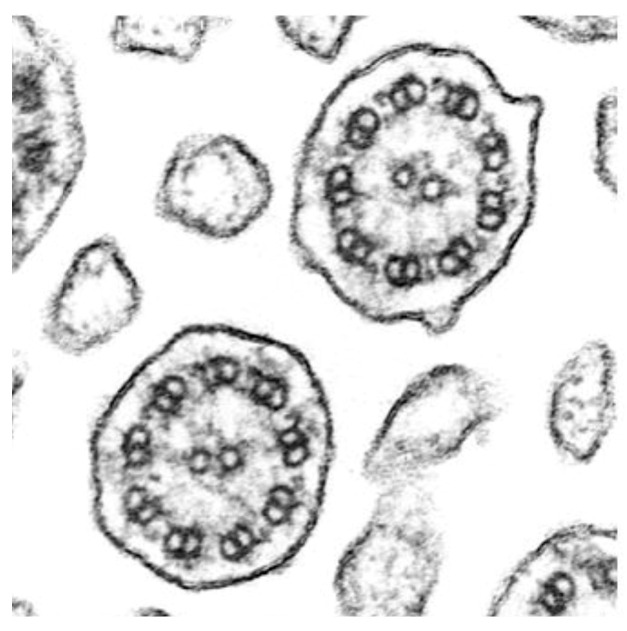
Ciliary sections obtained from post-transplant biopsy study (bronchial brushing). All ciliary sections show preserved ultrastructure.

The second patient, who underwent transplantation a year later, was assessed at 3, 6, 12, and 18 months post-transplant, with findings similar to the first patient; the nasal cilia remained dyskinetic, while the bronchial ciliary function was normal (See Supplementary Video S3), suggesting stable bronchial ciliary function in the transplanted lungs. The mean ciliary beat frequency in the bronchial epithelial samples was 9.6 Hz, with a range of 8.5–12 Hz. Importantly, transmission electron microscopy analysis of the biopsy samples from the transplanted lungs in both patients showed normal ciliary ultrastructure (Figure 5).

## 4. Discussion

In patients with PCD undergoing lung transplantation, preserving the functionality of bronchial mucociliary clearance is crucial for ensuring the long-term success of the transplanted organ. Understanding whether the respiratory ciliated epithelium retains the donor’s functional characteristics or if the recipient’s genetic alterations impact ciliary function is essential.

The results of our study demonstrated that, at 30 months post-transplant for patient 1 and 18 months for patient 2, the bronchial cilia of the transplanted lungs maintained normal motility. These findings suggest that lung transplantation can preserve normal ciliary function in the lower airways of the transplanted organ, despite the patient’s genetic alterations causing ciliary dysfunction in other tissues. This is an encouraging sign for both graft survival and the patient’s prognosis. The transplanted lung functions as an “island” within the body, free from the recipient’s genetic defect.

These results are consistent with the previous clinical studies demonstrating the benefits of lung transplantation in patients with advanced-stage PCD and progressive lung deterioration [14,15,16,18,19,20,21]. However, we did not find any prior studies in the literature specifically evaluating ciliary function in the transplanted lungs of patients with PCD. It is noteworthy that a recent multicenter study, which represents the largest cohort to date of lung transplant recipients with PCD, retrospectively collected data from 36 patients who underwent lung transplantation between 1995 and 2020. The findings confirmed that, despite surgical challenges associated with laterality disorders in some patients, lung transplantation is a viable therapeutic option for patients with PCD with end-stage disease. However, this study did not include specific assessments of ciliary motility and ultrastructure in the bronchial epithelium, relying instead on clinical evaluation through pulmonary function tests, survival analysis, and the absence of chronic lung allograft dysfunction.

These findings are promising for PCD research and suggest that lung transplantation can be a feasible therapeutic option in this population. Additionally, they provide a foundation for future investigations and improvements in therapeutic approaches for this disease. Nevertheless, additional studies with larger sample sizes and longer follow-up periods are needed to confirm and generalize these results.

The main limitation of our study is the small sample size. However, given the rarity of the disease, the low number of patients with PCD requiring lung transplantation, and the consistency of findings in both cases, we believe these results provide valuable clinical insights.

## 5. Conclusions

This study contributes new scientific knowledge on PCD and provides relevant insights into the impact of lung transplantation on ciliary function in these patients. The results support the hypothesis that lung transplantation preserves bronchial ciliary motility, a critical factor for long-term transplant success. These findings establish a solid foundation for further research and continued improvement in therapeutic strategies for patients with PCD.

## Data Availability

The data in the study can be obtained by contacting the corresponding author.

## Supplementary Materials

The following supporting information can be downloaded at: https://www.mdpi.com/article/10.3390/jcm14103439/s1, Video S1: Bronchial cells from a transplanted lung with normal cilliary motility, 6 months post-transplant. Video S2: Bronchial cells from a transplanted lung with normal cilliary motility, 12 months post-transplant. Video S3: Bronchial cells from a transplanted lung with normal cilliary motility, 18 months post-transplant.

## Abbreviations

The following abbreviations are used in this manuscript:

PCD	Primary Ciliary Dyskinesia
